# Casitas B-lineage lymphoma linker helix mutations found in myeloproliferative neoplasms affect conformation

**DOI:** 10.1186/s12915-016-0298-6

**Published:** 2016-09-08

**Authors:** Lori Buetow, Giancarlo Tria, Syed Feroj Ahmed, Andreas Hock, Hao Dou, Gary J. Sibbet, Dmitri I. Svergun, Danny T. Huang

**Affiliations:** 1 Cancer Research UK Beatson Institute, Garscube Estate, Switchback Road, Glasgow, G61 1BD UK; 2 EMBL c/o DESY, Notkestrasse 85, Geb, 25a, 22603 Hamburg, Germany; 3 Present address: Institute of Medical Genetics, School of Medicine, Shandong University, No. 44 Wenhuaxi Road, Jinan, Shandong 250012 People’s Republic of China; 4 Present address: Multimodal Molecular Imaging Institute, Nanoscopy Division, Maastricht University, Universiteitssingel 50, 6229 ER Maastricht, The Netherlands

**Keywords:** Ubiquitin, Cbl, Myeloproliferative neoplasms, SAXS, Transformation potential

## Abstract

**Background:**

Casitas B-lineage lymphoma (Cbl or c-Cbl) is a RING ubiquitin ligase that negatively regulates protein tyrosine kinase (PTK) signalling. Phosphorylation of a conserved residue (Tyr371) on the linker helix region (LHR) between the substrate-binding and RING domains is required to ubiquitinate PTKs, thereby flagging them for degradation. This conserved Tyr is a mutational hotspot in myeloproliferative neoplasms. Previous studies have revealed that select point mutations in Tyr371 can potentiate transformation in cells and mice but not all possible mutations do so. To trigger oncogenic potential, Cbl Tyr371 mutants must perturb the LHR-substrate-binding domain interaction and eliminate PTK ubiquitination. Although structures of native and pTyr371-Cbl are available, they do not reveal how Tyr371 mutations affect Cbl’s conformation. Here, we investigate how Tyr371 mutations affect Cbl’s conformation in solution and how this relates to Cbl’s ability to potentiate transformation in cells.

**Results:**

To explore how Tyr371 mutations affect Cbl’s properties, we used surface plasmon resonance to measure Cbl mutant binding affinities for E2 conjugated with ubiquitin (E2–Ub), small angle X-ray scattering studies to investigate Cbl mutant conformation in solution and focus formation assays to assay Cbl mutant transformation potential in cells. Cbl Tyr371 mutants enhance E2–Ub binding and cause Cbl to adopt extended conformations in solution. LHR flexibility, RING domain accessibility and transformation potential are associated with the extent of LHR-substrate-binding domain perturbation affected by the chemical nature of the mutation. More disruptive mutants like Cbl Y371D or Y371S are more extended and the RING domain is more accessible, whereas Cbl Y371F mimics native Cbl in solution. Correspondingly, the only Tyr371 mutants that potentiate transformation in cells are those that perturb the LHR-substrate-binding domain interaction.

**Conclusions:**

c-Cbl’s LHR mutations are only oncogenic when they disrupt the native state and fail to ubiquitinate PTKs. These findings provide new insights into how LHR mutations deregulate c-Cbl.

**Electronic supplementary material:**

The online version of this article (doi:10.1186/s12915-016-0298-6) contains supplementary material, which is available to authorized users.

## Background

Dysregulated signalling is a prominent feature in cellular transformation and tumorigenesis. The proto-oncogene Casitas B-lineage lymphoma (Cbl or c-Cbl), encodes an E3 ubiquitin ligase that downregulates PTK-directed cell signaling through ubiquitination, thereby targeting these kinases for lysosomal or proteasomal degradation [1, 2]. Cbl is a member of the Cbl family of proteins, so characterized based on a highly conserved N-terminal region that contains the structural components required for ubiquitin ligase activity. In simpler eukaryotic organisms, such as *Caenorhabditis elegans* and *Dictyostelium discoideum*, only one Cbl protein is present, but in mammals there are three, including Cbl, Cbl-b and Cbl-c.

**Fig. 1:**
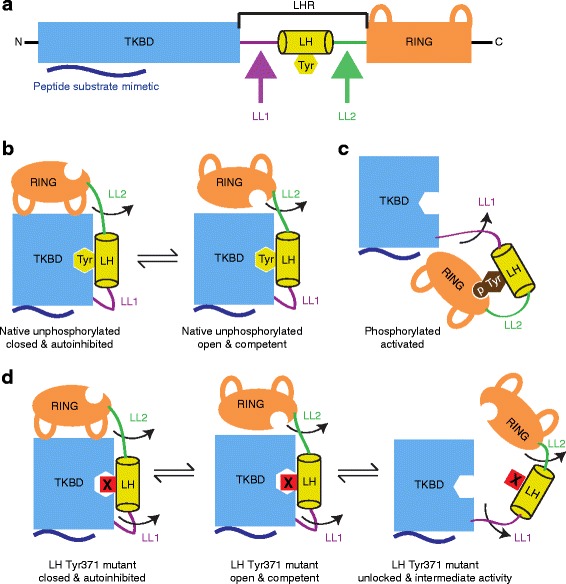
Model depicting linker helix (LH)-mediated regulation of Cbl ligase. **a** Domains of Cbl’s N-terminal ubiquitin ligase region. A *blue rectangle* and *navy wavy line* correspondingly depict the TKBD and its substrate-binding site. The LHR and its components are indicated with LL1 coloured purple, LL2 green and LH yellow. A *yellow hexagon* depicts the LH Tyr. The RING is coloured orange with the two loops representing the E2-binding site. **b** Model of unphosphorylated Cbl coloured as in (**a**). When Tyr371 is unphosphorylated, the LH is clamped to the TKBD and movement of the RING restricted to the TKBD face opposing the substrate-binding site. Rotation about LL2 allows Cbl to fluctuate between an autoinhibited state where the E2-binding site is occluded (*left*) and a catalytically competent state where E2 is able to bind (*right*). **c** Model of phosphorylated Cbl coloured as in (**a**). A *brown hexagon* and *circle* represent the phosphorylated LH Tyr. When Tyr371 is phosphorylated, the LH clamps into the RING domain and LL1 rotates 180°, juxtaposing the RING domain and substrate-binding face of the TKBD. The Tyr371 binding site on the TKBD cannot accommodate a phosphate moiety, thereby eliminating the LH-TKBD interaction. Whether the RING domain is flexible in solution is unknown. **d** Model depicting possible states of Cbl Tyr 371 mutants coloured as in (**a**). The LH Tyr371 mutant is shown as an X in a *red square*. Cbl Y371X mutants are predicted to favour different states depending on the chemical nature of the substitution. Less disruptive mutants are expected to maintain the unphosphorylated equilibrium (*left* and *middle*), whereas more disruptive mutants are expected to perturb the LH-TKBD interaction, leaving the RING in an open, catalytically competent conformation with greater access to space surrounding the TKBD (*right*)

The conserved N-terminus of Cbl family proteins contains a substrate tyrosine kinase-binding domain (TKBD), a linker helix region (LHR) and a RING domain (Fig. 1a). The TKBD confers specificity to Cbl’s ligase activity based on the selective recruitment of phosphorylated substrates containing an (N/D)XpY(S/T)XXP, DpYR or RA(V/I)XNQpY(S/T) motif [3]. The RING domain mediates the transfer of ubiquitin (Ub) from an E2 Ub-conjugating enzyme to the substrate [4, 5]. Within the LHR is a conserved tyrosine (Tyr371 in Cbl) that is crucial for regulating ligase activity. Phosphorylation of this tyrosine enhances ligase activity and is essential for ubiquitination of receptor PTKs [6–11]. In addition to the highly conserved N-terminus, Cbl and Cbl-b also have extensive C-termini that confer adaptor-like functions to these proteins based on the ability to mediate multiple protein-protein interactions. These include a proline rich region that mediates interactions with SH3 domain-containing proteins and a tyrosine rich region that, upon phosphorylation, becomes a binding motif for other SH2 domain-containing proteins [12]. Cbl and Cbl-b terminate with an ubiquitin-associated domain, which is crucial for homo- and heterodimerization of these two Cbl proteins [13, 14].

Cbl abnormalities are associated with a number of human cancers. High expression of Cbl is commonly observed in several human breast cancer cell lines as well as primary breast and prostate cancer tissues [15, 16]. In addition, Cbl mutations are observed in myelodysplastic syndromes-myeloproliferative neoplasms (MDS/MPN) and non-small cell lung cancers [17, 18]. Cbl is suggested to have different roles in pathological processes depending on its function as a ligase or adaptor. Breast and prostate cancer cell line oncogenic characteristics are reduced when Cbl is knocked down, and, in breast cancer cells, independent of its ligase activity, Cbl mediates suppression of transcriptional activity induced by the tumour suppressor transforming growth factor-β1 [15, 16]. In contrast, more in-depth studies of select MDS/MPN mutants suggest that these mutants potentiate transformation because they fail to ubiquitinate and thereby downregulate PTK signalling [19, 20].

The highest frequency of missense mutations in MDS/MPN clinical samples occurs at Tyr371; identified mutations include Cbl Y371S, Y371H, Y371C, and Y371D. Cbl Y371S and Y371H have been investigated extensively in independent studies [19, 20]. Cbl Y371S promotes colony formation in soft-agar assays and induces tumour formation when injected into nude mice. Likewise, Cbl Y371H promotes cytokine-independent growth in cells but only when endogenous Cbl is knocked down. Both display defects in their ability to ubiquitinate the PTK EGFR. For Tyr371 mutations in the LHR, although defective PTK ubiquitination contributes to aberrant cell signalling, it is not sufficient to potentiate transformation. Cbl Y371F shows reduced EGFR ubiquitination in vitro and in cells [6, 21], but it is significantly less efficient in promoting colony formation in soft agar assays and does not induce tumour formation when injected into mice [21, 22].

A number of factors contribute to how phosphorylation of Tyr371 regulates Cbl’s ligase activity. In the unphosphorylated state, Cbl’s RING domain rotates between an open, catalytically competent conformation and a closed conformation (Fig. 1b) [5, 6]. In the closed conformation, the E2-binding site on the RING domain is occluded and Cbl is autoinhibited. Although the RING can bind E2 thioesterified with Ub (E2 ~ Ub; ~ indicates thioester bond) in the unphosphorylated state, the RING domain is restricted to the TKBD face opposite the substrate-binding site by interactions between the LHR and TKBD. The LHR comprises a long loop (called linker loop 1 or LL1, residues 353–363) followed by an α-helix (LH, residues 364–374) and then a second shorter loop (LL2, residues 375–380). Tyr371 is on the LH, and, in the unphosphorylated state, Tyr371 and several other residues from the LH, including Tyr368, bind to the TKBD, limiting movement between the RING and TKBD to rotation about LL2. Phosphorylation of Tyr371 eliminates this LHR-TKBD interaction – modelling suggests the Tyr371 binding pocket on the TKBD is too small to accommodate a phosphate group. Instead, pTyr371-LHR forms a new set of interactions with the RING domain that stabilizes E2 ~ Ub during Ub transfer [6, 7]. Movement within LL2 is more restricted by this new set of interactions but a conformational change in LL1 juxtaposes the RING domain with the substrate-binding face of the TKBD (Fig. 1c). In solution, whether LL1 is flexible and adopts multiple conformations or prefers the crystallographically observed conformation where the RING is juxtaposed with the substrate binding face of the TKBD is unclear.

We postulate that Y371 mutations might have different oncogenic potentials due to flexibility differences in Cbl’s linker region mediated by the LHR-TKBD interaction (Fig. 1d). We predict that, while a large aromatic residue like Phe cannot duplicate the Tyr371-TKBD interactions, neither will it perturb those interactions; hence, Cbl Y371F can adopt and readily maintain conformations observed in the unphosphorylated state. On the other hand, mutations, such as Y371S, introduce a polar residue into a hydrophobic pocket and are likely to perturb or occlude the LH-TKBD interaction, thereby unlocking the LHR from the TKBD and leaving Cbl in an intermediate activity state where the E2 ~ Ub binding site on the RING domain is exposed but lacking the pTyr371-LHR E2 ~ Ub stabilizing component (Fig. 1c,d). To investigate the influence of mutations and phosphorylation on Cbl’s LHR flexibility and RING accessibility in solution, we performed small-angle X-ray scattering (SAXS) analysis and surface plasmon resonance (SPR) assays on pTyr371-Cbl, unphosphorylated Cbl and a selection of Cbl variants. We then performed focus formation assays to explore the relationship between this flexibility and oncogenic potential.

## Results

### Cbl’s LHR-TKBD interactions affect RING binding affinity for E2 ~ Ub

Previous studies suggest Cbl Y371 mutants have the potential to perturb or abrogate LHR-E2 interactions essential for ligase activity [5, 21]. To investigate this possibility, we used SPR to measure binding of the substrate E2 UbcH5B conjugated to Ub (UbcH5B–Ub; hyphen indicates a non-hydrolyzable amide linkage) to variants of the N-terminal fragment of Cbl comprising the TKBD, linker region and RING domain (N-Cbl, residues 47–435), as performed previously [6, 23]. We tested the unphosphorylated N-Cbl variants containing the Tyr371 mutations identified in MDS/MPN clinical samples as well as N-Cbl Y371A, which behaves similarly to the MDS/MPN Tyr371 mutants [21], and N-Cbl Y371F based on its reduced transformation potential in cells and mice. In addition, we tested Cbl Y371E, which has previously been shown to stimulate Cbl activity in vitro [8]. N-Cbl Y368F was also included in our assays because this mutation is required to generate homogeneously phosphorylated pTyr371-N-Cbl; previous studies demonstrate this mutation does not affect the structure or ligase activity in vitro or in cells [6, 21, 22]. We also included N-Cbl M222E, which disrupts the closed conformation without perturbing the TKDB-LHR interaction [6], so that we could investigate changes induced by rotation about LL2. We predicted that, if the RING domain were unfolded, no binding of UbcH5B–Ub to N-Cbl Y371 variants is expected, whereas, if these variants simply weaken the LHR-TKBD interaction, Cbl Y371 mutants will adopt the closed conformation less frequently and thus bind UbcH5B–Ub more tightly than wild-type N-Cbl. None of the N-Cbl Y371 mutants is expected to bind UbcH5B–Ub as tightly as pTyr371-N-Cbl because pTyr371 eliminates the closed, autoinhibited conformation and forms additional interactions with UbcH5B ~ Ub. All of the N-Cbl variants in our SPR assay bind UbcH5B–Ub with similar or higher affinity than wild-type N-Cbl (84 μM) but more weakly than pTyr371-N-Cbl (2.6 μM, Table 1), suggesting mutations in the LHR do not affect the competency of the RING domain to bind UbcH5B–Ub. Other mutations in the LHR also affect LHR-TKBD interactions as evidenced by the higher binding affinity of N-Cbl Y368F (49 μM) for UbcH5B–Ub than wild-type N-Cbl.

**Table 1:** Dissociation constants (*K*_d_) for interactions between Cbl variants and UbcH5B–Ub

Immobilized GST-Cbl variant	Analyte	*K*_d_ (μM)
WT	UbcH5B–Ub	84 ± 2
Y371F	UbcH5B–Ub	84 ± 2
Y371H	UbcH5B–Ub	39 ± 1
Y371C	UbcH5B–Ub	36 ± 1
Y371A	UbcH5B–Ub	30 ± 1
Y371S	UbcH5B–Ub	16 ± 0.7
Y371D	UbcH5B–Ub	10 ± 0.5
Y371E	UbcH5B–Ub	9 ± 0.4
pY371 Y368F	UbcH5B–Ub	2.6 ± 0.6
Y368F	UbcH5B–Ub	49 ± 2
M222E	UbcH5B–Ub	14 ± 0.6

Standard errors of the mean are indicated

The SPR data demonstrate that the chemical characteristics of the side chain of the mutated amino acid in the N-Cbl Y371 variants influence binding affinity for UbcH5B–Ub: the greater potential the mutation has to perturb the Tyr371 binding pocket on the TKBD, the higher the binding affinity for UbcH5B–Ub. The highest binding affinities are observed when Tyr is substituted with an amino acid that has a shorter, non-aromatic and polar or charged side chain like N-Cbl Y371S (16 μM), Y371E (9 μM), and Y371D (10 μM); the binding affinities of these mutants are comparable to N-Cbl M222E (14 μM), which disrupts the RING-TKBD interaction in the closed, autoinhibited conformation. In contrast, when Tyr371 is substituted with Phe, the UbcH5B–Ub binding affinity is comparable to wild-type (N-Cbl Y371F, 84 μM) and the LHR-TKBD interaction is unperturbed, as demonstrated by the crystallographic structure of N-Cbl Y371F (Additional file 1: Figure S1, Table S1). For the remaining MDS/MPN mutants, polar and aromatic or short and hydrophobic amino acids are substituted for Tyr371 (N-Cbl Y371H, Y371A and Y371C). These are expected to weaken the interactions within the Tyr371 binding pocket but not as effectively as the group of mutants with short and polar or charged side chains. Thus, the UbcH5B–Ub binding affinity of these mutants is expected to fall between wild-type and the more disruptive mutants and this is indeed what we observe in our SPR assays: N-Cbl Y371H (39 μM), Y371A (30 μM) and Y371C (36 μM).

### Characterization of wild-type and pTyr371 Cbl by SAXS

**Fig. 2:**
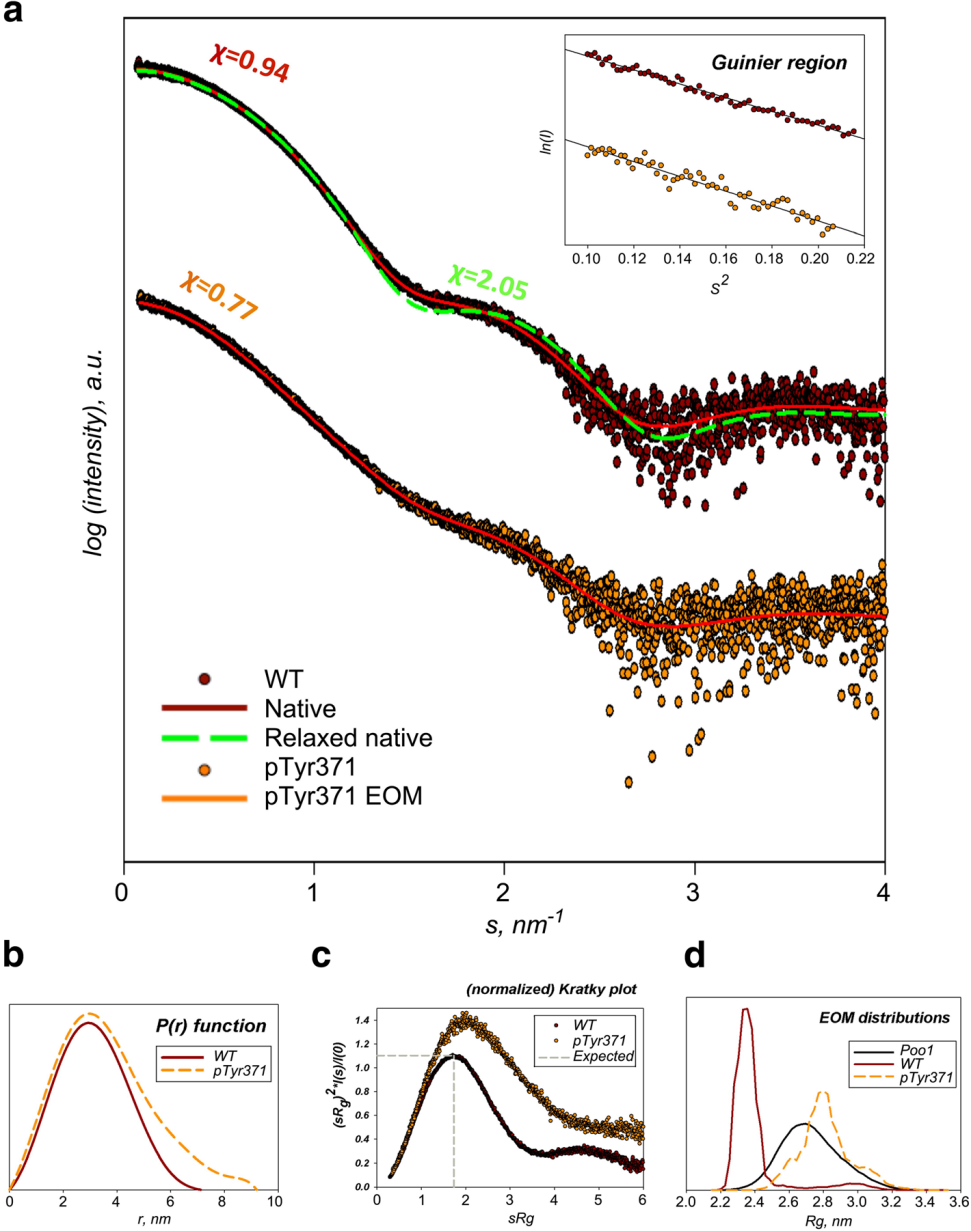
SAXS analysis of wild-type (WT) and pTyr371-N-Cbl. **a** *Top*: WT experimental scattering data (*dark red*) versus theoretical scattering curve from crystal structure (PDB:2Y1M) before (*dashed green line*; χ = 2.05) and after normal modes refinement (*solid red line*; χ = 0.94). *Bottom*: pTyr371-N-Cbl experimental scattering data (*orange*) versus theoretical scattering from the EOM 2.0 ensemble (*orange line*; χ = 0.77). **b** *P(r)* distribution of WT (*solid red*) and pTyr371-N-Cbl (*dashed orange*). **c** Normalized Kratky plot of WT and pTyr371-N-Cbl coloured as in (**a**). **d** EOM 2.0 distributions of the random pool (*black*) and the ensembles of WT and pTyr371-N-Cbl coloured as in (**b**)

Initially, we sought to investigate how Tyr371 phosphorylation affects the conformation of Cbl in solution by performing SAXS analysis on pTyr371-N-Cbl and wild-type N-Cbl. The monomeric state of these N-Cbl variants was confirmed by the SAXS-derived overall parameters (Fig. 2, Table 2, Additional file 1: Table S2) and gel filtration chromatography (data not shown). The deduced molecular masses (MMs) ranged from 41 to 48 kDa and are comparable with a predicted monomer mass of ~45 kDa for N-Cbl.

**Table 2:** SAXS parameters for wild-type N-Cbl and pTyr371-N-Cbl

Parameter	Wild-type	pTyr371
*R*_g_ [*P(r)*]	2.4 nm	2.8 nm
*R*_g_ [Guinier]	2.4 nm	2.8 nm
*D*_max_	7.0 nm	9.2 nm
*V*_Porod_	66.5 nm^3^	71.5 nm^3^
*V*_Dammif_	86.5 nm^3^	95.5 nm^3^
MM_Porod_	43 kDa	46 kDa
MM_Dammif_	44 kDa	47 kDa

*Rg* radius of gyration, *D*_*max*_ maximum particle distances, *V *volume, *MM* molecular mass

The SAXS-derived radius of gyration (*R*_g_) of wild-type N-Cbl variant is ~2.4 nm whereas pTyr371-N-Cbl is ~2.8 nm, and the calculated maximum particle distances (*D*_max_) are ~7.0 and ~9.2 nm, respectively. These differences indicate significant conformational changes in N-Cbl upon phosphorylation of Tyr371. The presence of an extended tail in the *P*(*r*) function of pTyr371-N-Cbl (Fig. 2b, Additional file 1: Figure S2) suggests rearrangements of the RING domain that make the particle more elongated as compared to the native one. In addition, the normalized Kratky plots (Fig. 2c) for wild-type and pTyr371-N-Cbl demonstrate significant differences. The wild-type reveals the peak at s *R*_g_ ≈ √3, as expected for globular particles [24], whereas for pTyr371-N-Cbl the peak position is significantly shifted suggesting the presence of structural disorder.

**Fig. 3:**
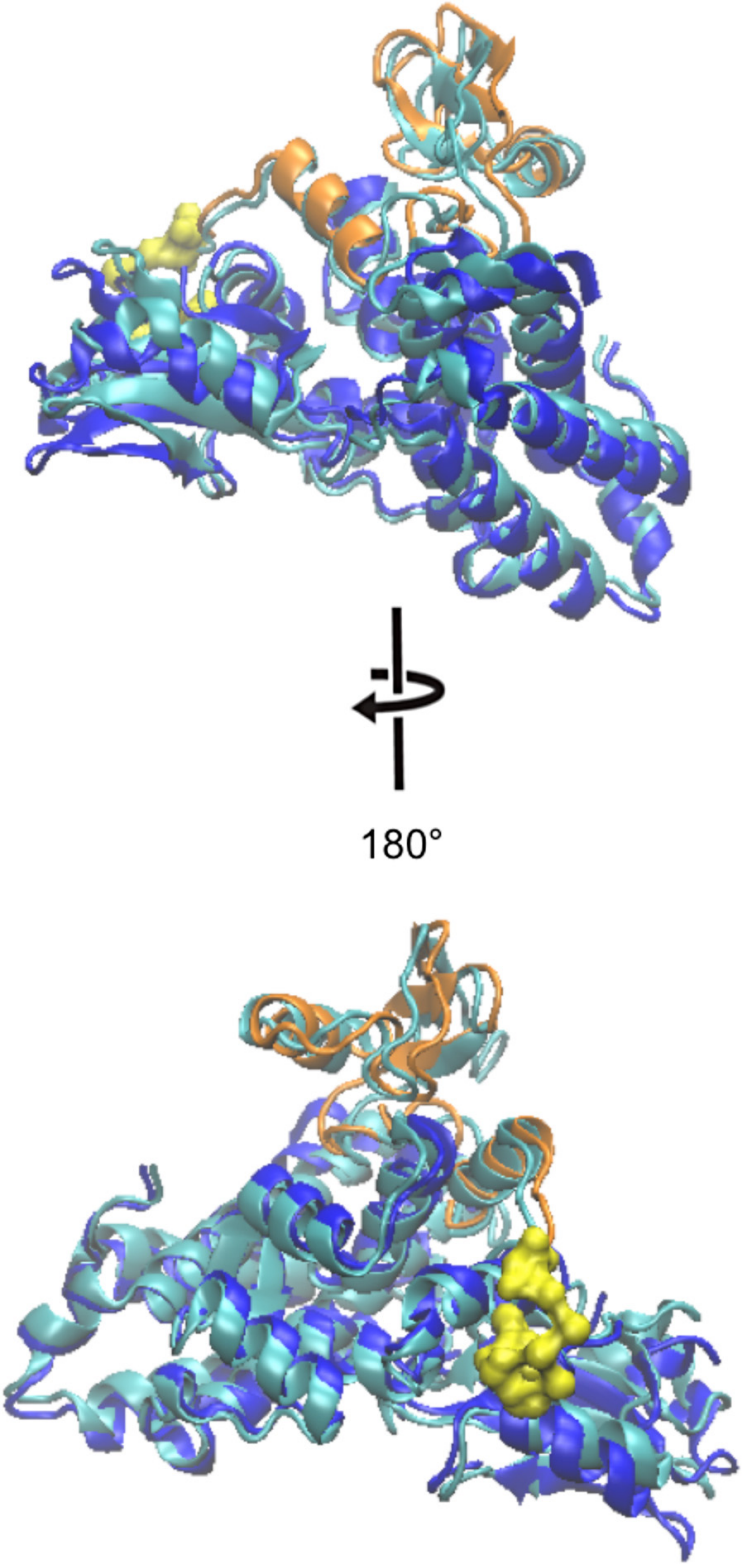
Comparison of crystal structure of N-Cbl (PDB 2Y1M) and the model derived from iterative normal mode analysis. The unphosphorylated, catalytically competent N-Cbl from the crystal structure (PDB 2Y1M) is coloured *cyan* and is superposed with the normal mode analysis-refined model. The TKBD is shown in *blue*, the RING domain in *orange* and the LHR in *yellow* as a surface model

The theoretical scattering computed from the native, unphosphorylated crystal structure (PDB:2Y1M) gives an overall reasonable fit to the experimental data but also displays some systematic deviations (Fig. 2a). These deviations point to possible differences between solution and crystalline states of the native protein. The crystal structure was therefore refined using an iterative normal mode analysis [25]. This procedure yielded a model which was similar to the original structure (root mean square deviation 1.015 Å) and provided a significant improvement of the fit from χ = 2.05 to 0.95. Ab initio shape reconstruction further confirms the globular state of unphosphorylated Cbl (Fig. 3, Additional file 1: Figure S3).

**Fig. 4:**
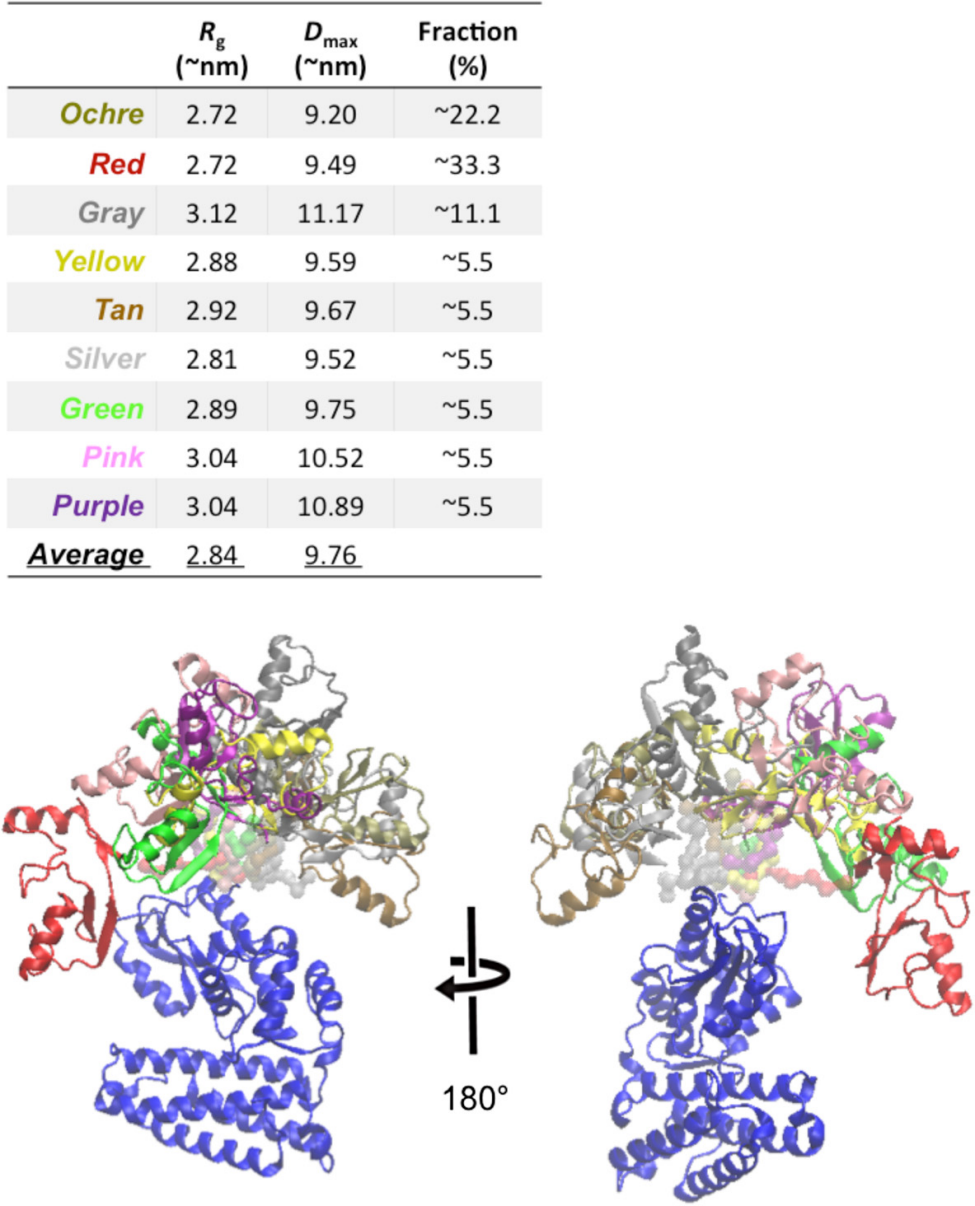
Cartoon ensemble representation of pTyr371-N-Cbl. The TKBD domain is coloured *blue*. The predicted RING domain conformations are shown in several colours as indicated in the table. The missing loops connecting the RING and TKBD domains are coloured according to the corresponding RING domains but presented as transparent surfaces

Given the expected flexibility of pTyr371-N-Cbl, structural modelling was performed using an Ensemble Optimization Method (EOM) [45]. Here, ensembles of models with variable conformations are selected from a pool of randomly generated models such that the scattering from the ensemble fits the experimental data, and the distributions of the overall parameters (e.g. *R*_g_) in the selected pool are compared to the original pool. As a negative control, EOM was first used on the data from N-Cbl and the selected ensembles showed predominantly compact conformations (Fig. 2d). In contrast, the selected ensembles for pTyr371-N-Cbl displayed broader distributions of predominantly extended conformations, which were on average more extended than the random pool (Fig. 2d). These data indicate that pTyr371-N-Cbl is extended and flexible in solution, suggesting that phosphorylation of the LH allows the RING domain to adopt multiple conformations in solution and access multiple surfaces of the TKBD (Fig. 4).

### Characterization of N-Cbl mutants by SAXS

To study how LHR-TKBD interactions influence the flexibility of LL1 in solution, we performed SAXS on a concentration series of our N-Cbl Tyr371 mutants, N-Cbl Y368F and N-Cbl M222E. The monomeric state of all N-Cbl variants was confirmed by the SAXS-derived overall parameters (Additional file 1: Table S2) and gel filtration chromatography (data not shown) with the exception of N-Cbl Y371C, where strong concentration-dependent effects were observed; for this reason, N-Cbl Y371C was omitted from further SAXS analysis. For the remaining N-Cbl variants, the deduced MMs ranged from 41 to 48 kDa pointing to a monomeric state in solution.

**Fig. 5:**
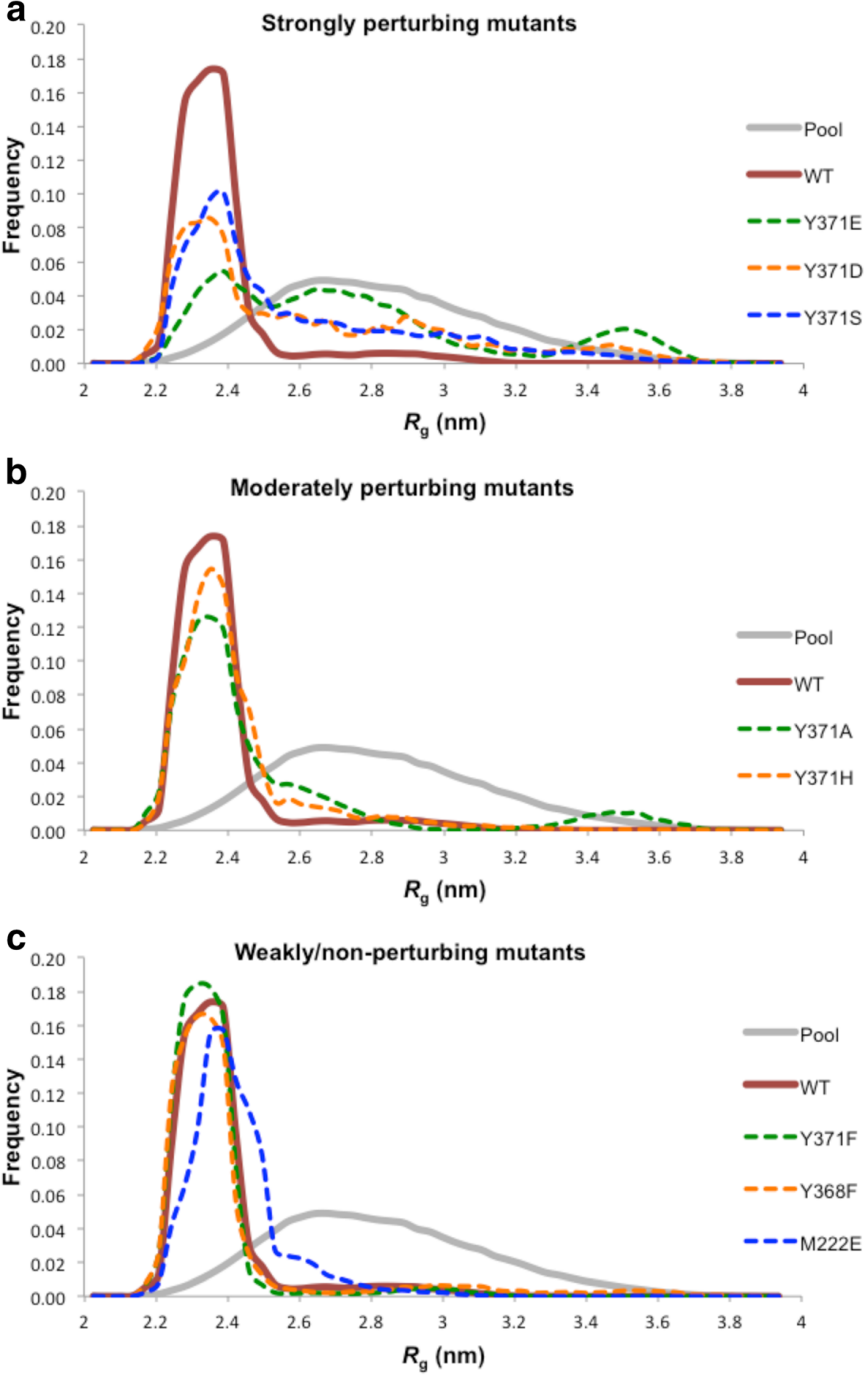
EOM 2.0 analysis of N-Cbl Tyr371 mutants, N-Cbl Y368F and N-Cbl M222E. Mutants were clustered according to their ability to perturb the LHR-TKBD interaction based on SAXS parameters shown in Table 3. **a** Strongly perturbing mutants. **b** Moderately perturbing mutants. **c** Weakly or non-perturbing mutants

Comparison of the SAXS-derived parameters as well as quantification of the flexibility (*R*_flex_) estimated by using EOM revealed a link between the conformation of N-Cbl in solution and the extent of perturbation of the Tyr371-TKBD interaction similar to the trend observed in our SPR data (Tables 1 and 3 and Fig. 5). Our N-Cbl Y371 variants were classified into four categories, depending on the extent of LHR-TKBD perturbation observed based on our SPR findings: complete (pTyr371-N-Cbl), strongly-perturbing (Y371S, Y371D, Y371E), moderately perturbing (Y371A, Y371H, Y371C), and non-perturbing (Y371F). pTyr371-N-Cbl, which abolishes the LHR-TKBD interaction, appears the most elongated, whereas unphosphorylated, wild-type N-Cbl appears more compact. Compared to wild-type N-Cbl, the strongly perturbing mutants, N-Cbl Y371S, Y371E and Y371D, are partially elongated (*R*_g_s are 2.6, 2.7 and 2.7 nm, respectively) but not as much as pTyr371-N-Cbl. Conformational rearrangements of these mutants are also confirmed by the corresponding *R*_flex_ values of approximately 84 %, 90 % and 88 % compared to approximately 60 % for wild-type N-Cbl. The moderately and non-perturbing mutants have *R*_g_ and *D*_max_ values more similar to the wild-type N-Cbl, although with different degrees of flexibility (Table 3 and Fig. 5). Notably, N-Cbl Y371F appears slightly more compact and less flexible than wild-type N-Cbl, with an *R*_g_ of 2.38 nm, a *D*_max_ of 7.67 nm, and an *R*_flex_ of approximately 56 %. Together with SPR, these data demonstrate that, in solution, RING domain accessibility is connected to N-Cbl conformational rearrangements induced by mutation or modification of Tyr371 in the LHR.

**Table 3:** Average ensemble outcome *R*_g_ and *D*_max_ from EOM 2.0 together with the χ value and the quantification of flexibility (*R*_flex_) for each N-Cbl variant

N-Cbl variant	*R*_g_ (nm)	*D*_max_ (nm)	Fit (χ value)	*R*_flex_ (%)
Y371F	2.38	7.67	1.284	56
WT	2.41	7.89	0.831	60
Y368F	2.46	8.37	0.778	65
M222E	2.44	8.05	1.030	65
Y371H	2.44	8.24	0.695	69
Y371A	2.52	8.16	1.020	75
Y371S	2.61	8.89	0.774	84
Y371D	2.64	8.91	0.814	88
Y371E	2.79	9.43	0.773	90

The Tyr371-TKBD interaction appears to play a critical role in mediating how elongated N-Cbl is in solution. Although N-Cbl Y368F and M222E have higher binding affinities for UbcH5B–Ub than wild-type, at SAXS resolution both have characteristics comparable to wild-type, suggesting they are similarly compact in solution. Although N-Cbl M222E has a comparable binding affinity to the strongly-perturbing N-Cbl Y371 mutants, in solution, it appears more compact, as both *R*_g_ and *D*_max_ are similar to wild-type N-Cbl and flexibility is relatively limited (*R*_flex_ ~60 %). N-Cbl Y368F has a higher binding affinity for UbcH5B–Ub than wild-type, but at the level of resolution provided by SAXS its characteristics and compactness are similar to those of the wild-type protein, suggesting similar behaviour in solution.

### Transformation potential of Cbl Y371 mutants in cells

**Fig. 6:**
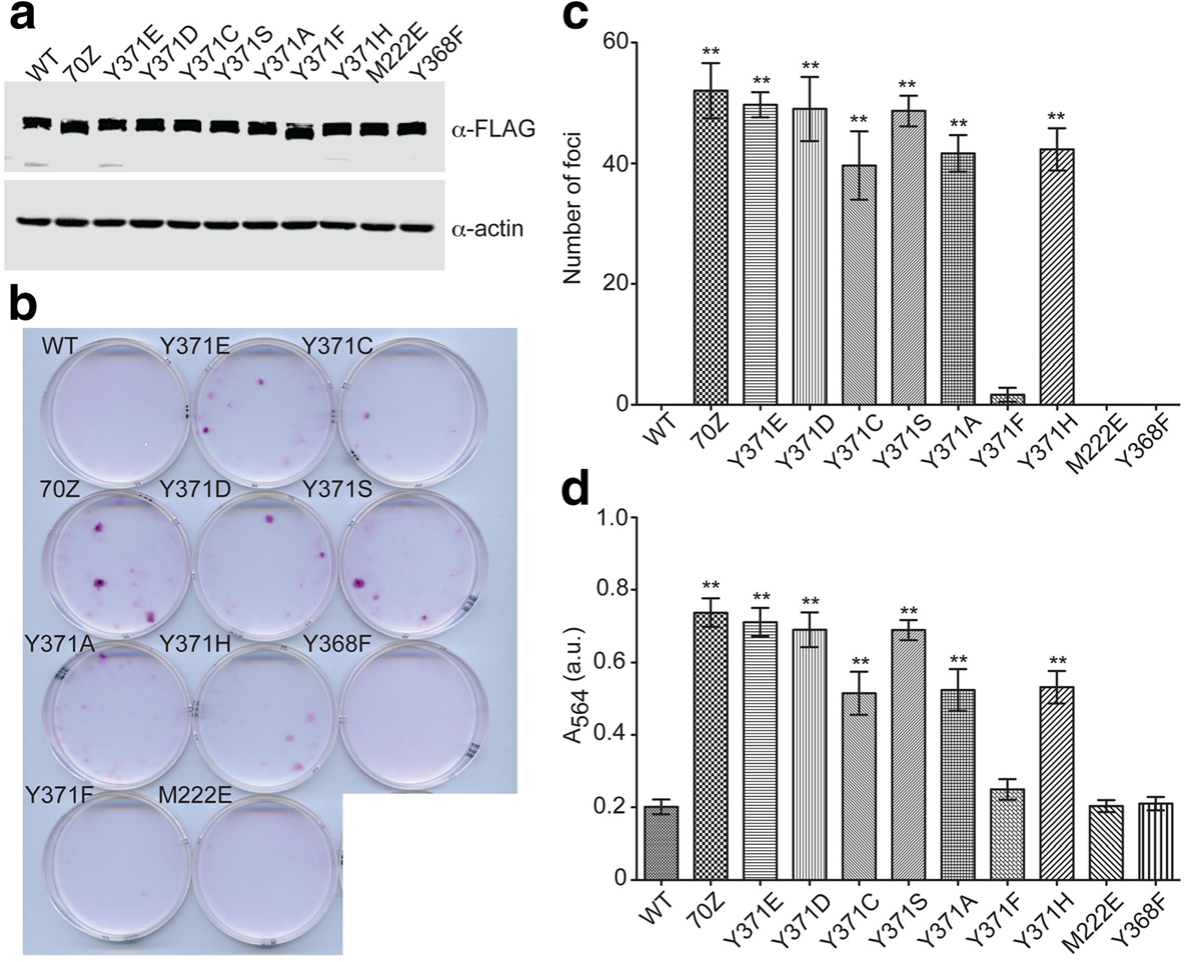
Transformation potential of Cbl variants in focus formation assays. **a** Immunoblot of lysates from 3T3 fibroblasts infected with FLAG-tagged Cbl variants using α-FLAG antibody (*top*) and α-actin (*bottom*) antibody as a loading control. **b** Sulforhodamine B-stained 3T3 fibroblasts infected with indicated Cbl variants. **c** Mean number of foci formed by Cbl variant-infected 3T3 fibroblasts shown in a bar graph (*n* = 3). No foci were present in 3T3 cells infected with wild-type Cbl, Cbl M222E or Cbl Y368F. Double asterisks (**) denote significant differences (*P* < 0.05) between indicated Cbl variant and wild-type using ANOVA followed by Dunnett’s multiple comparisons test. Error bars indicate standard deviation. **d** As in (**c**) but for A_564_ of extracted sulforhodamine B from Cbl-infected 3T3 fibroblasts

To test the transforming potential of Cbl Y371 mutants in cells, we performed focus formation assays using 3T3 cells stably transfected with N-terminally FLAG-tagged Cbl variants. Relative protein expression levels of each variant were assessed by immunoblotting (Fig. 6a). Foci were visualized with sulforhodamine B staining and counted manually with a DNA Safe Imager (Invitrogen). Afterwards, the sulforhodamine B stain was extracted and the A_564_ measured to compare relative cell densities [26, 27]. Foci counts and relative cell densities were analyzed by one-way ANOVA followed by Dunnett’s test with wild-type Cbl as the control. Cbl70Z was included among the variants tested as a positive transforming control [22]. Other variants tested included the same set used in our biochemical assays except in a full-length context.

Foci were observed in cells transfected with Cbl Y371 mutants and Cbl70Z, but not in wild-type, Cbl Y368F- or Cbl M222E-transfected cells (Fig. 6). When analyzed by one-way ANOVA followed by Dunnett’s test, significant differences (*P* < 0.05) in number of foci and cell densities were observed between cells infected with wild-type Cbl and all the Y371 mutants except Cbl Y371F (Fig. 6c,d, Additional file 1: Figure S4). Significantly more foci were present in Cbl70Z, Cbl Y371E and the MDS/MPN Cbl Y371 mutant set and the relative cell density was significantly greater compared to wild-type Cbl; however, there is no correlation between mutations that promote moderate or strong LHR-TKBD perturbations and transformation potential. Based on the ability to form foci, all the Cbl variants except Cbl Y368F and M222E have the ability to potentiate transformation in cells. Based on statistical analyses of the number of foci and relative cell density, Cbl70Z, Cbl Y371E and the MDS/MPN Cbl Y371 mutant set have significantly higher numbers of foci than wild-type Cbl. For Cbl Y371F, neither the number of foci nor the relative cell density are significantly different than in cells infected with wild-type Cbl; these results suggest this mutant may potentiate transformation but not to the same extent as the other Cbl Y371 mutants or Cbl70Z.

## Discussion

In this work, we employed SAXS and SPR to characterize how modifications and mutations in Cbl affect the conformation and accessibility of the RING domain in solution and subsequently investigate the relationship between these characteristics and the ability of Cbl variants to potentiate transformation in cells. Our results show that a mutation (M222E) within the RING-TKBD interface regulates accessibility of the RING domain only, whereas mutation or modification of Tyr371 within the LHR mediates the LHR-TKBD interaction, thereby regulating RING domain accessibility as well as the space sampled by the RING domain relative to the TKBD. The chemical nature of the substitution or modification controls the LHR-TKBD interaction and shifts the equilibrium between the native conformation where the RING is restricted to the face of the TKBD opposing the substrate-binding site and open conformations where the RING domain can access other surfaces of the TKBD and potentially other regions of Cbl. None of the Tyr371 mutants can adopt the pTyr371 conformation that is critical for activation of E2 ~ Ub and RTK ubiquitination, but all of the Tyr371 mutants perturb the LHR-TKBD conformation observed in the native state except Cbl Y371F. Likewise, all of the Tyr371 mutants except Cbl Y371F clearly potentiate transformation in our focus formation assays, whereas Cbl Y371F forms foci but statistical analyses of the number of foci formed and relative cell densities suggest that this mutant has less transformation potential than the others. Previous studies have shown that Cbl Y371F is not transforming in soft agar colony formation assays nor does it promote tumour formation in nude mice [21, 22]. These data indicate that the flexibility of the LHR features in the Cbl Tyr371 mutant transformation mechanism.

Met222 is a key residue in maintaining the RING-TKBD interaction in native Cbl. A Met to Glu mutation was used to perturb this interaction and investigate the importance of the RING-TKBD interaction on Cbl’s conformation in solution. Our previous work and SPR studies show that mutation of Met222 in the TKBD to Glu increases Cbl’s E2–Ub binding affinity by approximately 8-fold and enhances Cbl’s in vitro autoubiquitination rate [6]. Though Cbl M222E has a higher binding affinity for UbcH5B–Ub and is more active than wild-type Cbl, SAXS analysis demonstrates that Cbl M222E has parameters (*R*_g_, *D*_max_ and *R*_flex_) comparable to wild-type Cbl, suggesting both adopt a compact conformation. In cells, Cbl M222E does not enhance EGFR ubiquitination (data not shown) nor does it potentiate transformation in our focus formation assays. The lack of effect of this mutant in these assays suggests structural regulation of Cbl’s ligase activity requires more than a simple rotation that exposes the E2-binding site on the RING domain.

Previous work has shown that phosphorylation of the conserved LHR Tyr in the Cbl family enhances activity in vitro and is essential for receptor PTK ubiquitination in cells, where Cbl’s E3 ligase activity features prominently in EGFR downregulation via lysosomal degradation [8, 10, 11]. The rate and pattern of Cbl-mediated EGFR ubiquitination determines whether EGFR is directed to the lysosome or recycled to the membrane. X-ray crystallography and NMR studies of Cbl and Cbl-b have shown that this phosphorylation event eliminates RING-mediated autoinhibition, optimally positions Ub for transfer and juxtaposes the RING domain with the TKBD substrate-binding site [6, 7, 9]. Notably, in the crystal structures of Cbl and Cbl-b bound to Zap70 peptide and E2 or E2 ~ Ub, the conformations of RING domain relative to the TKBD are identical. Here, we show that, in solution, when Tyr371 is phosphorylated, the *R*_g_ and *D*_max_ increase and EOM analysis demonstrates the linker region becomes flexible, allowing the RING domain to sample the space surrounding the TKBD rather than maintain the crystallographically observed conformation. This is consistent with other E3 ligases, where translational and rotational movement of the RING domain are required for Ub transfer to select substrate lysine sites (monoubiquitination and multiubiquitination) as well as polyUb chain formation [28]. Cbl binds EGFR directly through its TKBD and indirectly through growth factor receptor binding 2-mediated interactions in the region C-terminal to the RING domain [12]. Cbl-mediated ubiquitination of EGFR is observed for both binding sites, highlighting a potential essential role for RING flexibility in Cbl substrate ubiquitination.

Our SAXS and SPR data show that mutations within the LHR perturb the native LHR-TKBD conformation and that the extent of perturbation is dependent on the chemical nature of the amino acid substitution. In the native state, Trp258, Ala262, Val263, and Met274 form a hydrophobic pocket around the aromatic ring of Tyr371 and the side chain of Ser227 forms a hydrogen bond with the hydroxyl group of Tyr371. The more disruptive the mutation is to the hydrophobic environment, the greater the perturbation in the LHR-TKBD interaction. When Tyr371 is mutated to an amino acid with a charged side chain like Asp or Glu or a small, polar side chain like Ser, the UbcH5B–Ub binding affinity is approximately 8-fold enhanced compared to native Cbl and the *R*_g_ and *D*_max_ increase to values comparable to pTyr371-Cbl. Additionally, SAXS analysis reveals these Tyr371 mutants are the most flexible compared to native Cbl. Mutation of Tyr371 to smaller hydrophobic amino acids like Cys or Ala only moderately perturbs the LHR-TKBD interaction as evidenced by an approximately 2-fold enhancement in the UbcH5B–Ub binding affinity and the moderate increase in *R*_g_, *D*_max_ and *R*_flex_ compared to native Cbl. Likewise, when Tyr371 is mutated to His, only moderate to weak perturbation of the LHR-TKBD interaction occurs. There is an approximately 2-fold enhancement in the UbcH5B–Ub binding affinity and SAXS analysis shows evidence of more flexibility (slightly higher values of *R*_g_, *D*_max_ and *R*_flex_) compared to native Cbl. When Tyr371 is mutated to Phe, the hydrophobic interactions are not disturbed and the native state is maintained: the UbcH5B–Ub binding affinity, *R*_g_, *D*_max_ and *R*_flex_ values are comparable to native Cbl. In addition, previous in vitro autoubiquitination assays show native Cbl and Cbl Y371F have comparable catalytic efficiencies [6]. Thus, in solution, it appears as though Cbl Y371F mimics the behaviour of native Cbl.

Mutation of Tyr371 is not the only site at which the LHR-TKBD interaction can be perturbed. Our previous work and current data show that mutation of Tyr368 to Phe also disrupts the LHR-TKDB interaction [6]. Cbl Y368F binds UbcH5B–Ub approximately 2-fold more tightly than native Cbl and has an approximately 2-fold enhancement in catalytic efficiency for in vitro autoubiquitination. In addition, this mutant displays a slight increase in all SAXS-based parameters (including *R*_flex_) compared to native Cbl. However, in contrast to the Cbl Tyr371 mutants, Cbl Y368F does not promote focus formation in our assays nor does it compromise EGFR ubiquitination in cells [6] and previous work has also shown this mutant does not form colonies in soft agar assays nor promote tumour growth in nude mice [21, 22]. Though it is clear that Tyr368-TKBD interactions also play a role in the conformations Cbl adopts in solution, the significance of this interaction in Cbl’s ligase activity in cells is unclear. Other studies have shown that Cbl ΔY368 is defective in EGFR ubiquitination and has the ability to potentiate transformation in cells and in mice [21, 22]. In addition, Cbl Y368C has also been identified in MDS/MPN clinical samples, and, like Cbl Y371C and Y371S, has been shown to potentiate transformation in cells and in mice; however, whether PTK ubiquitination is compromised remains unknown [19]. As observed for Tyr371, the chemical nature of the mutation at Tyr368 might factor into regulation of Cbl’s ligase activity as well as transformation potential.

While the ability to ubiquitinate PTKs is essential for Cbl-mediated downregulation of PTK signalling, previous work has clearly shown that it is not the only prerequisite for Cbl-dysfunction driven transformation. Thien et al. [21] propose that the LHR-TKBD stability of Cbl mutants contributes to their ability to potentiate transformation based on the analysis of a number of linker region and RING mutants: Cbl ΔY368, ΔY371 and Y371A promote transformation but not Cbl Y368F or Y371F. Cbl Y368F is slightly more flexible than native Cbl but retains the ability to ubiquitinate receptor PTKs, whereas Cbl Y371F cannot ubiquitinate receptor PTKs but is able to maintain the native conformation in solution. Thus, it seems that both characteristics contribute to the transformation potential of Cbl mutants. Cbl functions as both an ubiquitin ligase and adaptor in receptor PTK-mediated cell signalling. Although enzymatic activity is slower in the absence of Tyr371 phosphorylation, Cbl is still functional [6]. It may be that when the LHR-TKBD interaction is perturbed, Cbl ubiquitinates substrates that bind to regions other than the canonical TKBD-binding site or the more flexible mutants might ubiquitinate “dead end” lysines on substrates such that biologically viable ubiquitination chains are not formed. Alternatively, perturbed LHR-TKBD interactions might disrupt oligomerization or interactions with other proteins or lead to the non-sequential recruitment of substrates to exposed surfaces that interfere with controlled signalling.

## Conclusions

We have shown that LHR-TKBD interactions regulate Cbl’s ligase activity and conformations in solution. Mutations or modifications within the LHR prevent Cbl from maintaining a closed conformation where the RING domain is restricted to a surface opposite the TKBD-substrate binding site; instead, depending on the nature of the alteration, Cbl becomes flexible and the RING domain can access multiple surfaces of the TKBD. Transformation only occurs when mutations within the LHR (1) perturb the native state and (2) fail to ubiquitinate PTKs.

## Methods

### Protein preparation

For crystallization, N-Cbl Y371F (residue 47–435) was expressed, purified and stored as described previously [6]. For SPR and SAXS analysis, N-Cbl variants were cloned into a modified form of pGEX4T1 (GE Healthcare) containing an N-terminal histidine glutathione S-transferase (His-GST) tag followed by a thrombin or TEV-protease cleavage site and expressed in *E. coli* BL21 (DE3) Gold. pTyr371-N-Cbl was generated as described previously [6]. For SPR analysis, all N-Cbl variants were purified by Ni-NTA followed by glutathione-affinity chromatography. Anion exchange chromatography was subsequently used to separate His-GST-pTyr371-N-Cbl from unphosphorylated His-GST-N-Cbl. For SAXS studies, N-Cbl variants were then treated with TEV or thrombin protease to cleave the His-GST tag and further purified by Ni-NTA pass-back followed by anion exchange and size exclusion chromatography. Proteins for SAXS were stored in 25 mM Tris-HCl (pH 7.6), 500 mM NaCl and 1 mM DTT at –80 °C. UbcH5B S22R C85K–Ub (referred to as UbcH5B–Ub) was expressed, generated and purified as described previously [7]. His-GST-tagged protein concentrations were determined by Bradford assay using BSA as a standard and all other concentrations were determined using a NanoVue Spectrophotometer (GE Healthcare).

### Crystallization and structural determination

Crystals were obtained by mixing N-Cbl Y371F (10 mg/mL) with an equal volume of reservoir solution containing 0.1 M Tris-HCl, pH 8.5, 3.0–3.1 M sodium formate, and 5 mM DTT using hanging drop vapour diffusion at 8 °C. The crystals were flash-frozen in 0.1 M Tris-HCl, pH 8.5, 3.0–3.1 M sodium formate, 5 mM DTT, 8 % (v/v) glycerol, 8 % (v/v) ethylene glycol, and 8 % (v/v) sucrose. Data were collected with beamline I04 at Diamond Light Source, integrated with automated XDS [29] and scaled using the CCP4 program suite [30]. Initial phases were obtained by molecular replacement with PHASER using native N-Cbl (PDB:2Y1M) [6]. N-Cbl Y371F crystals belong to space group C222_1_ with six molecules in the asymmetric unit. The model was built in COOT [31] and refined using PHENIX [32].

### Biacore analysis

Cbl–UbcH5B–Ub binding experiments were conducted as described previously [6, 23]. GST-Cbl variants were coupled to CM-5 chips and binding was measured at a concentration range of 0–120 μM of UbcH5B–Ub. The data were analyzed using Biacore T200 Evaluation software package (Biacore Life Sciences) and Scubber2.0c (BioLogic Software).

### Plasmids and cell culture

N-terminally FLAG-tagged Cbl variants were generated by PCR amplification and ligation into a pBABE vector [33] by In-Fusion (Clontech) according to the manufacturer’s instructions. Subsequently, these Cbl variants were transiently transfected into Phoenix Eco cells using Lipofectamine 2000 (Thermo Fisher Scientific) according to the manufacturer’s instructions. Infection was performed in 3T3 (mouse fibroblast) cells followed by puromycin (2 μg/mL) selection. Cells were cultured in DMEM containing 20 mM glutamine and 10 % donor bovine serum in a 37 °C incubator at 5 % CO_2_.

### Focus formation assays and sulforhodamine B extraction

To perform the focus formation assays, 3T3 cells were seeded onto 10 cm^2^ dishes and infected with the Cbl variants on the following day. This was followed by a second round of infection after 24 hours and the media changed on the following day. After 3 days of selection with puromycin, the 3T3 cells were split and 0.5 × 10^5^ cells seeded onto 3.5 cm^2^ dishes and cultured for 18 days before being fixed in methanol and stained with sulforhodamine B. Foci were then counted manually with a DNA Safe Imager (Invitrogen). Afterwards, each dish was incubated with 1.1 mL of 10 mM Tris, pH 10.5 for 5 minutes with rocking at room temperature to extract sulforhodamine B and the absorbance of the extracted dye was measured at 564 nm. Prism (Graph Pad, Mac V5.0C) was used for statistical analyses (one-way ANOVA followed by Dunnett multiple comparisons testing with *P* < 0.05 treated as the cutoff for significant differences).

### Immunoblotting

After 1 week, total protein was isolated from one 3.5 cm^2^ dish of each Cbl variant in whole cell lysis buffer containing 50 mM Tris, pH 7.4, 150 mM NaCl, 1 mM EDTA, 1 % Igepal CA-630 (Sigma), and 10 % glycerol. Proteins were separated under reducing conditions using SDS polyacrylamide gel electrophoresis and transferred onto a nitrocellulose membrane (GE Healthcare Life Sciences). Blots were probed with rabbit anti-FLAG (Sigma Aldrich, F7425) and goat anti-actin (SantaCruz Biotechnology, sc-1616) antibodies, incubated with donkey anti-goat IRDye 800CW and goat anti-rabbit IRDye 680LT secondary antibodies (LI-COR Biosciences, 925_32214 and 925_68021), and visualized using an Odyssey CLx Imaging System (LI-COR Biosciences).

### Small angle X-ray scattering

Synchrotron X-ray scattering data of Cbl mutants were collected at EMBL P12 beamline (DESY, Hamburg) using a robotic sample changer [34]. All the mutants were measured in the same buffer (500 mM NaCl, 25 mM Tris-HCl (pH 7.6), 1 mM DTT) in a concentration series ranging from either 0.5–10 mg/mL (0.5, 1, 2, 5, and 10 mg/mL) or 0.5–6.6 mg/mL (0.5, 1, 1.6, 3.3, and 6.6 mg/mL). SAXS data were recorded at 10 °C using a PILATUS 2 M pixel detector (DECTRIS, Baden, Switzerland) at a sample-detector distance of 3.1 m and a wavelength of 0.15 nm. This setup covers a range of momentum transfer of 0.1 < *s* < 5 nm^–1^ (*s = 4π sin(θ)/λ*, where *2θ* is the scattering angle). Initially, the data were pre-processed using an automatic pipeline [35] and further analyzed using PRIMUS [36, 37]. The forward scattering *I(0)* as well as the *R*_g_ were calculated using the Guinier approximation assuming that, at very small angles (*s* < 1.3/*R*_g_), the intensity is represented as *I(s) = I(0)* · *exp(-(sR*_g_*)*^2^*/*3*)* [38]. Linearity in the Guinier region was used to exclude sample aggregation. The pair-distance distribution function *P(r)*, from which the *D*_max_ and *R*_g_ were estimated, was computed using GNOM [39]. Qualitative assessment of compactness versus structural disorder was made by transforming the scattering profiles in the so-called Kratky representation [*I*(*s*)*s*^*2*^ vs. *s*] [40] and its normalized version [(*sR*_g_)^2^*I(s)/I(0) vs. *sR*_g_] [41]. The MM was derived from (1) the excluded volume of the hydrated particle using the Porod invariant [36] and (2) the excluded volumes of the ab initio models. Ab initio models were created with DAMMIF [42] using low resolution data (*s* < 2 nm^–1^). The algorithm constructs bead models yielding a scattering profile with the lowest possible discrepancy (χ) to the experimental data while keeping beads interconnected and the model compact. Twenty independent ab initio reconstructions were performed for each Cbl mutant and then averaged using DAMAVER [43]. Superimpositions between ab initio reconstructions and available atomic models were made using the software SUPCOMB [44]. The flexibility was analyzed using EOM 2.0 [45] – an enhanced version of the EOM [46], which assumes coexistence of a range of conformations in solution for which an average scattering intensity fits the experimental data. In EOM 2.0, a pool of 10,000 independent models is created as first step with the aim to approximate the (otherwise infinite) conformational space for a protein exhibiting disorder. For each model in the pool the theoretical scattering curve is automatically computed with CRYSOL [47]. Afterwards, genetic algorithm (GA) is used to select ensembles with varying numbers of conformers (usually from 5 to 40) by calculating the average theoretical profile and fitting it to the experimental SAXS data. For each Cbl mutant, the GA was repeated 100 independent times and the ensemble with the lowest discrepancy reported as the best solution out of 100 final ensembles. Furthermore, 100 repetitions of GA allowed the computation of *R*_g_ and *D*_max_ distributions so that structural information about the flexibility could be extracted. Distributions with *R*_g_ average values above the *R*_g_ average values calculated for the pool are classified as extended whereas models with values below the average as compact. The width of the distribution is also used to derive the flexibility of the particle, whereby a narrow distribution indicates a rather rigid particle and broader distributions are associated with higher flexibility [46]. Using EOM 2.0, systematic quantification of the flexibility was made using the metric *R*_flex_ – which computes the Shannon information entropy of the distributions [45]. All the software used for the SAXS data analysis belongs to the ATSAS 2.5 package [36].

## Additional file

Additional file 1: Figure S1. Comparison of wild-type and N-Cbl Y371F. **Figure S2.** Comparison of SAXS scattering data for wild-type (WT, blue) and pTyr371-N-Cbl (yellow). **Figure S3.** Comparison of ab initio and relaxed crystal models. **Figure S4.** Confidence intervals (95 %) for the difference between group means. **Table S1.** Data collection and refinement statistics. (DOCX 15797 kb)

## Abbreviations

Cbl: Casitas B-lymphoma lineage
*D*_max_: Maximum particle distance
E2 ~ Ub: E2 conjugated to ubiquitin via a thioester
EOM: Ensemble optimization method
GA: Genetic algorithm
GST: Glutathione S-transferase
LH: Linker helix
LHR: Linker helix region
LL1: Linker loop 1
LL2: Linker loop 2
MDS/MPN: Myelodysplastic syndromes-myeloproliferative neoplasms
MM: Molecular mass
N-Cbl: N-terminal fragment of Cbl comprising residues 47–435
PTK: Protein tyrosine kinase
*R*_flex_: Quantification of protein flexibility estimated by EOM
*R*_g_: Radius of gyration
SAXS: Small angle X-ray scattering
SPR: Surface plasmon resonance
TK: Tyrosine kinase
TKBD: Tyrosine kinase binding domain
Ub: Ubiquitin
UbcH5B–Ub: Ubiquitin stably conjugated to UbcH5B via an isopeptide bond
WT: Wild-type
