# Supplementary material for: Casitas B-lineage lymphoma linker helix mutations found in myeloproliferative neoplasms affect conformation
Source: BMC Biol. 2016 Sep 8;14(1):76. doi: 10.1186/s12915-016-0298-6 (PMC5015263; doi:10.1186/s12915-016-0298-6)
Supplement: Additional file 1: Figure S1. — Comparison of wild-type and N-Cbl Y371F. Figure S2. Comparison of SAXS scattering data for wild-type (WT, blue) and pTyr371-N-Cbl (yellow). Figure S3. Comparison of ab initio and relaxed crystal models. Figure S4. Confidence intervals (95 %) for the difference between group means. Table S1. Data collection and refinement statistics. (DOCX 15797 kb) [file 12915_2016_298_MOESM1_ESM.docx]

**
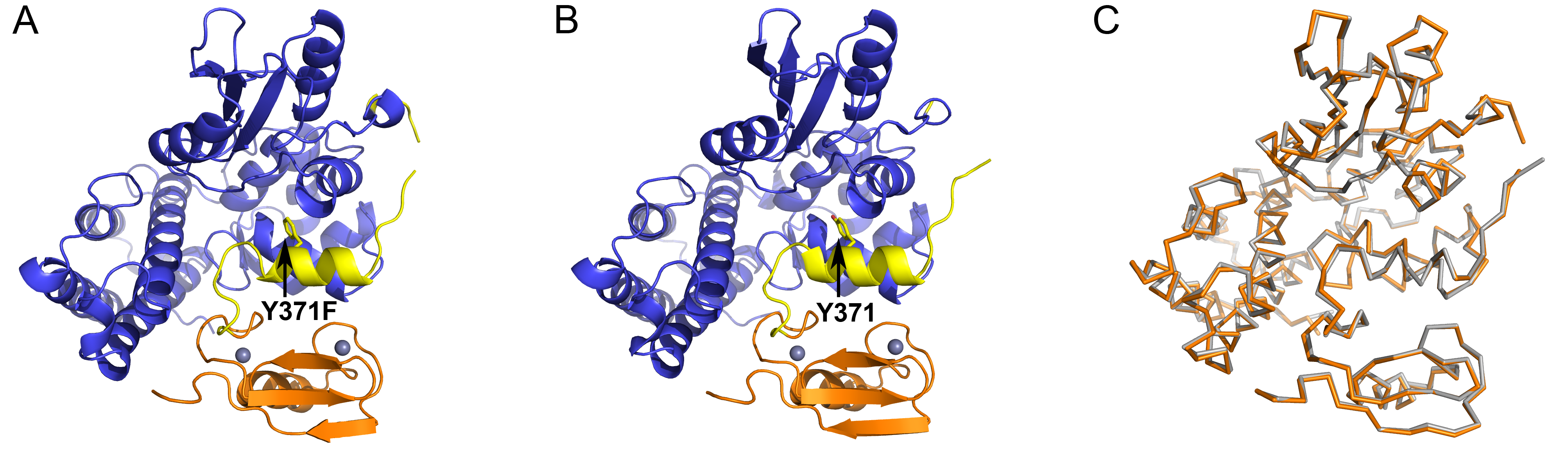
**

**Figure S1**. Comparison of wild-type and N-Cbl Y371F.

1. Cartoon diagram of the crystal structure of N-Cbl Y371F (PDB:XXXX) with the TKBD colored blue, the LHR yellow, and the RING domain orange. The Zn^2+^ ions are depicted as grey spheres. The Y371F substitution is shown in sticks and labelled.
2. Cartoon diagram of the crystal structure of wild-type N-Cbl (PDB:2Y1M) shown in the same orientation and colored as in (A).
3. Ribbon diagram of N-Cbl Y371F (orange) superposed onto wild-type N-Cbl (grey). The root mean square deviation between the two is 0.27 Å across 359 Cα atoms.


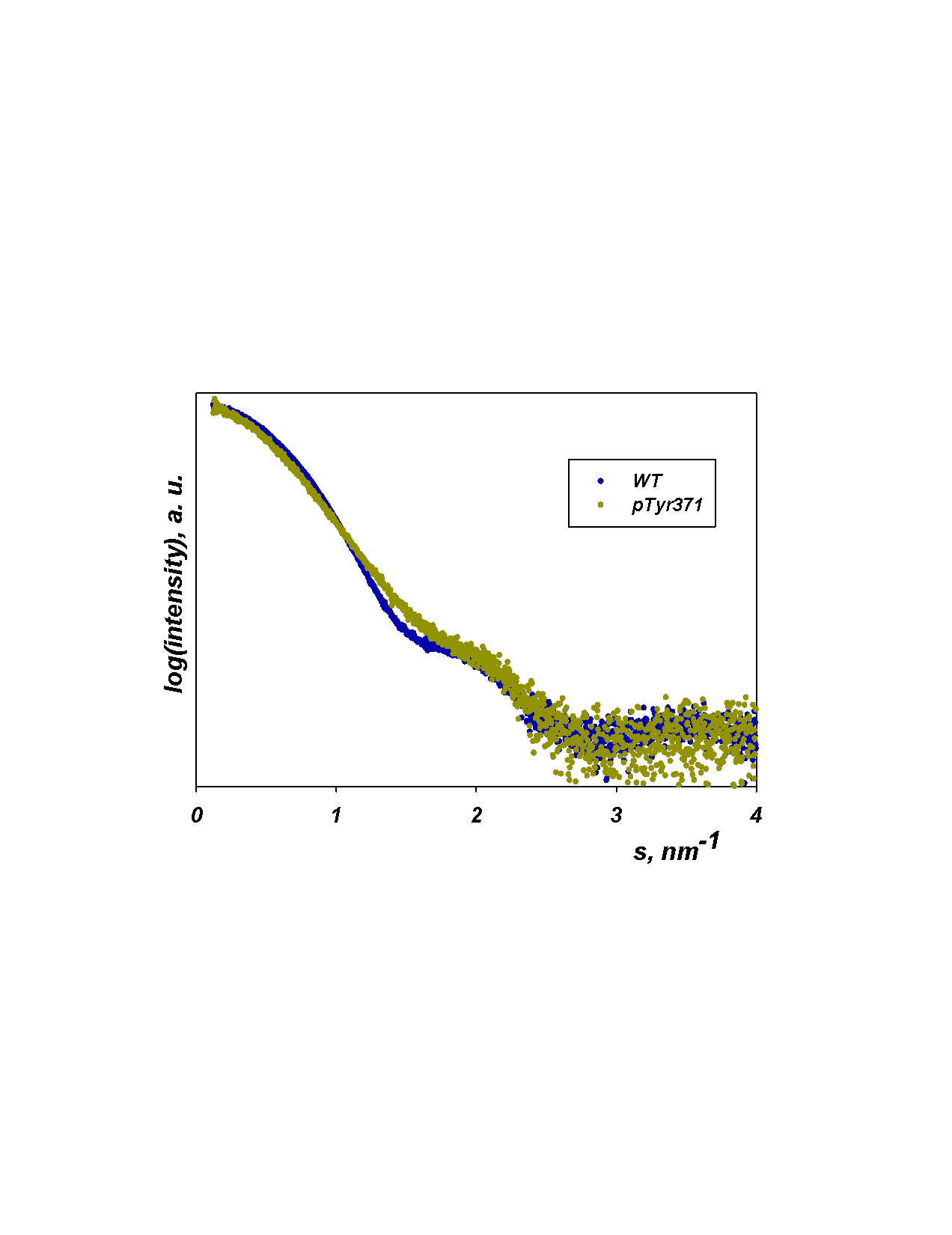


**Figure S2**. Comparison of SAXS scattering data for wild-type (WT, blue) and pTyr371-N-Cbl (yellow).


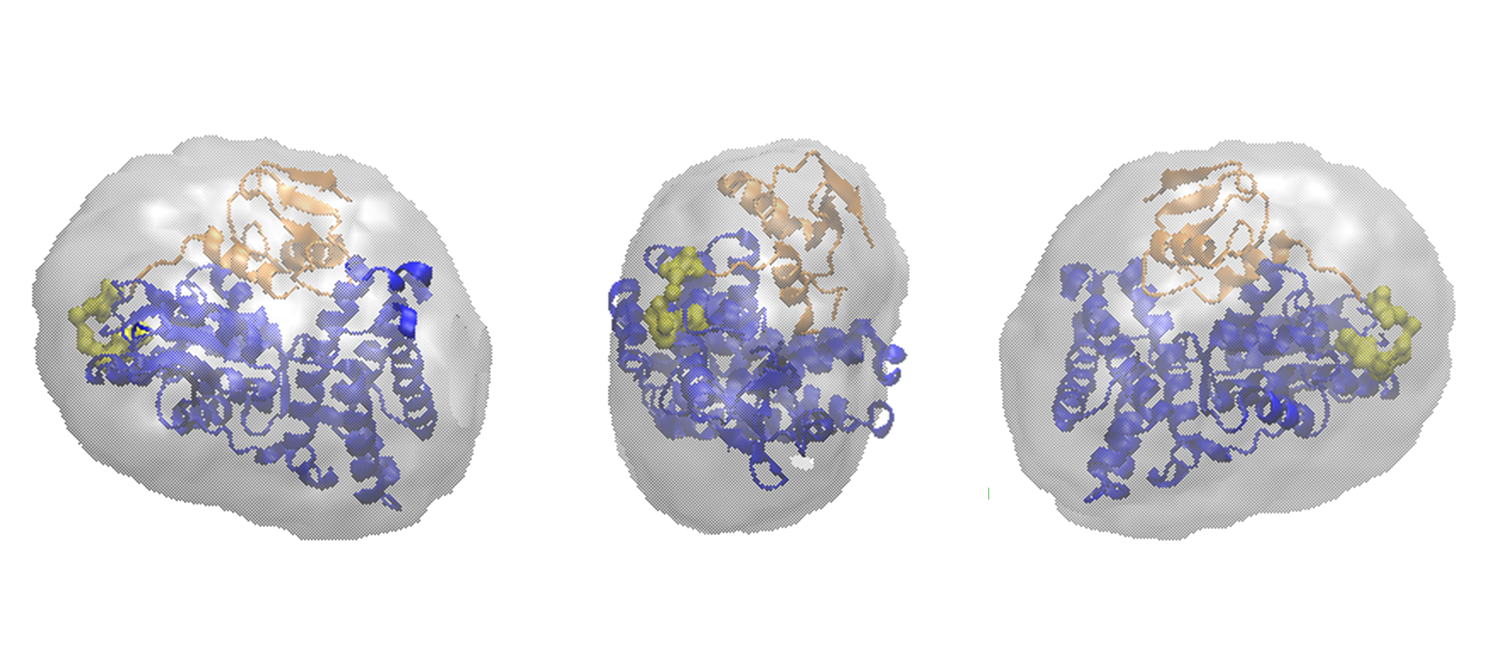

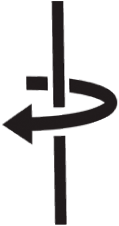


90°


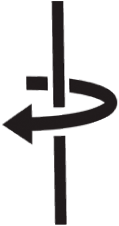


90°

**Figure S3**. Comparison of *ab initio* and relaxed crystal models.

Graphical superimposition of *ab initio* and relaxed crystal models

(PDB:2Y1M) of unphosphorylated N-Cbl with the crystal model shown as a cartoon. The TKBD and RING domain are colored blue and orange, respectively and the LHR is shown as a yellow space-filling model.

**
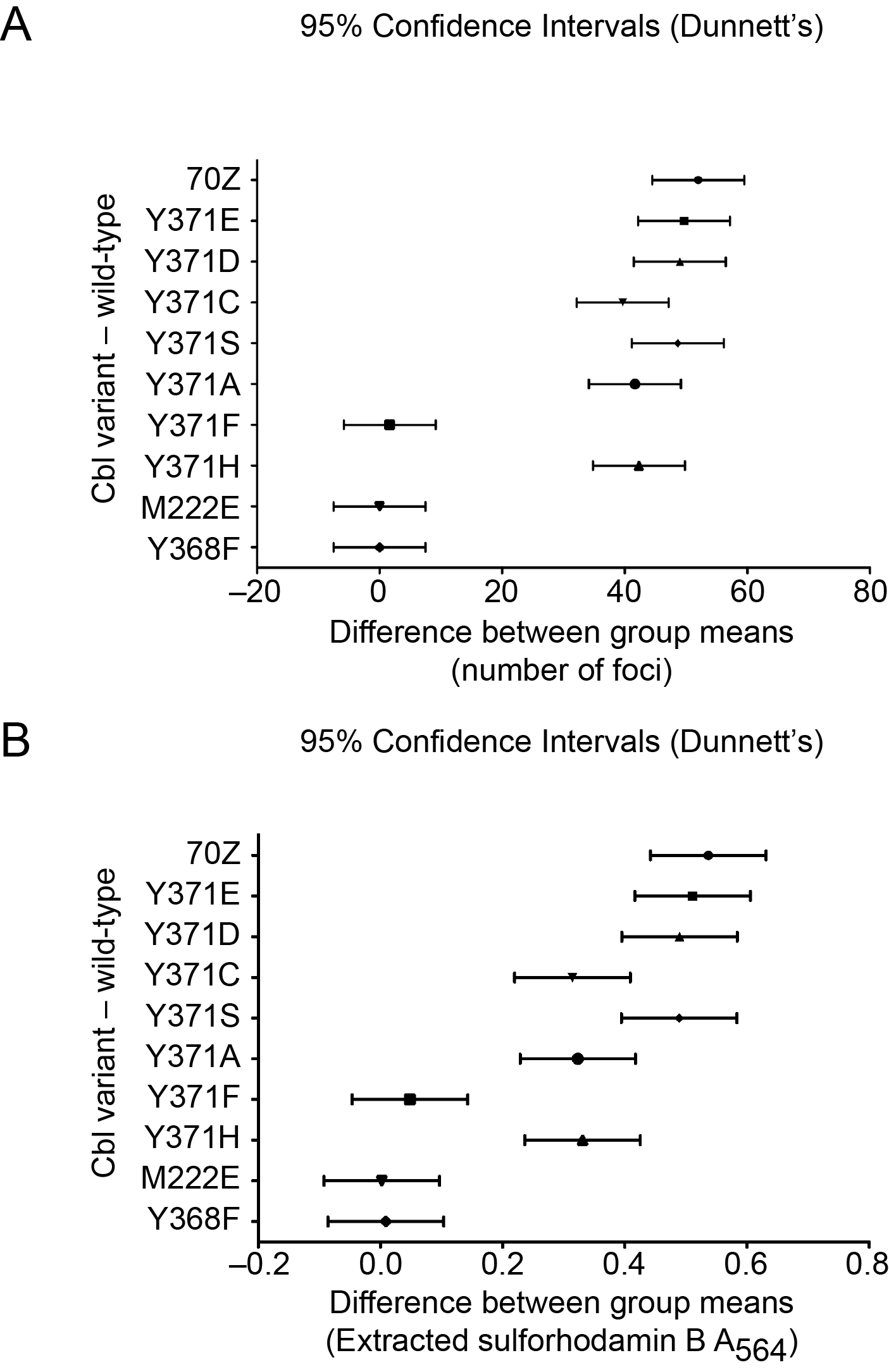
**

**Figure S4**. Confidence intervals (95%) for the difference between group means.

1. ANOVA followed by Dunnett’s test was used to compare the number of foci of indicated Cbl variants to wild-type Cbl (n=3). Symbols indicate the difference in the mean number of foci between each Cbl variant and wild-type Cbl and the lines surrounding each symbol denote the 95% confidence interval for the difference between the two means.
2. As in (A) but for the comparison between the A_630_ from extracted sulforhodamine B of indicated Cbl variants and wild-type Cbl.

**Table S1**

Data collection and refinement statistics

|  |  |
| --- | --- |
| **Data collection** |  |
| Space group | *C*222_1_ |
| Cell dimensions |  |
| *a*, *b*, *c* (Å) | 147.8, 149.0, 344.1 |
| *α* *β* *γ * (°) | 90, 90, 90 |
| Resolution (Å) | 105–2.82(2.90–2.82)^1^ |
| *R*_merge_ | 0.075(0.489) |
| *I* / σ*I* | 9.7(2.4) |
| Completeness (%) | 99.9(100.0) |
| Redundancy | 4.6(4.7) |
|  |  |
| **Refinement** |  |
| Resolution (Å) | 50.4–2.82 |
| No. reflections | 90968 |
| *R*_work_ / *R*_free_ | 0.212/0.254 |
| No. atoms |  |
| Protein | 18172 |
| Ligand/ion | 18 |
| Water | 94 |
| *B*-factors |  |
| Protein | 105.6 |
| Ligand/ion | 144.8 |
| Water | 80.5 |
| R.m.s. deviations |  |
| Bond lengths (Å) | 0.002 |
| Bond angles (°) | 0.54 |

^1^Values in parentheses are for highest-resolution shell.

| Table S2 | |  | | |  | |  |  |  | |  |
| --- | --- | --- | --- | --- | --- | --- | --- | --- | --- | --- | --- |
| SAXS statistics | |  | | |  | |  |  |  | |  |
|  | |  | | |  | |  |  |  | |  |
| Data collection parameters | |  | | |  | |  |  |  | |  |
|  | Instrument (detector) |  | | P12, EMBL, PETRA III  PILATUS 1M pixel (67 x 420 mm^2^) | | | | | | |  |
|  | Beam geometry |  | | 0.2 x 0.06 mm^2^ | | | | | | |  |
|  | Wavelength (Å) |  | | 1.5 | | | | | | |  |
|  | *s* range (Å^-1^) |  | | 0.012-0.6 | | | | | | |  |
|  | Temperature (K) |  | | 283 | | | | | | |  |
| Software employed | | |  | | |  | | | |  | |
|  | Primary data reduction |  | | RADAVER | | | | | | |  |
|  | Data processing |  | | PRIMUS | | | | | | |  |
|  | *Ab initio* analysis |  | | DAMMIF | | | | | | |  |
|  | Validation and averaging |  | | DAMAVER | | | | | | |  |
|  | Rigid body modeling |  | | BUNCH/CORAL/SASREF | | | | | | |  |
|  | Flexibility |  | | EOM | | | | | | |  |
|  | Computation of model intensities |  | | CRYSOL | | | | | | |  |
|  | 3D graphics representations |  | | VMD | | | | | | |  |
|  |  |  | |  | | | | | | |  |
| N-Cbl variant collection parameters | | ***WT*** | | | ***Y371F*** | | ***Y371H*** | ***Y371S*** |  |  |  |
|  | Concentration range (mg ml^-1^) | 0.5-10 | | | 0.5-10 | | 0.5-10 | 0.5-10 |  |  |  |
| Structural parameters | | | | |  | |  |  |  |  |  |
|  | *I*(0) (relative) [from *P*(*r*)] | 4986±10 | | | 5467±10 | | 4911±10 | 5058±10 |  |  |  |
|  | *R*_g_ (Å) [from *P*(*r*)] | 24±1 | | | 24±1 | | 24±1 | 26±1 |  |  |  |
|  | *I*(0) (relative) [from Guinier] | 5032±10 | | | 5479±10 | | 4957±10 | 5058±10 |  |  |  |
|  | *R*_g_ (Å) [from Guinier] | 24±1 | | | 24±1 | | 24±1 | 26±1 |  |  |  |
|  | *D*_max_ (Å) | 70±5 | | | 70±5 | | 70±5 | 90±10 |  |  |  |
|  | Porod volume estimate (Å^3^) | 66380±5000 | | | 69433±5000 | | 68862±5000 | 68383±5000 |  |  |  |
|  | Dammif excluded volume (Å^3^) | 86430±10000 | | | 92450±10000 | | 94810±10000 | 93540±10000 |  |  |  |
|  | Dry volume calculated from sequence (Å^3^) ‡ | ~54154 | | | ~54154 | | ~54154 | ~54154 |  |  |  |
| Molecular-mass determination | | | | | | |  |  |  |  |  |
|  | Molecular mass *M*_r_ (kDa) [from *Porod invariant*] | 40±5 | | | 42±5 | | 42±5 | 41±5 |  |  |  |
|  | Molecular mass *M*_r_ (kDa) [from *excluded volume*] | 44±5 | | | 46±5 | | 47±5 | 47±5 |  |  |  |
|  | Calculated monomeric *M*_r_ (kDa) [from *sequence*]* | ~44.7 | | | ~44.7 | | ~44.7 | ~44.7 |  |  |  |
| N-Cbl variant collection parameters | | ***Y371D*** | | | ***Y371C*** | | ***Y371A*** | ***Y371E*** |  |  |  |
|  | Concentration range (mg ml^-1^) | 0.5-10 | | | 0.5-10 | | 0.5-6.6 | 0.5-9 |  |  |  |
| Structural parameters | | | | |  | |  |  |  |  |  |
|  | *I*(0) (relative) [from *P*(*r*)] | 2963±15 | | |  | | 5683±15 | 6998±10 |  |  |  |
|  | *R*_g_ (Å) [from *P*(*r*)] | 27±1 | | |  | | 24±1 | 27±1 |  |  |  |
|  | *I*(0) (relative) [from Guinier] | 2965±15 | | |  | | 5707±15 | 6912±10 |  |  |  |
|  | *R*_g_ (Å) [from Guinier] | 27±1 | | |  | | 24±1 | 27±1 |  |  |  |
|  | *D*_max_ (Å) | 95±10 | | |  | | 74±5 | 90±10 |  |  |  |
|  | Porod volume estimate (Å^3^) | 67529±5000 | | |  | | 68383±5000 | 70111±5000 |  |  |  |
|  | Dammif excluded volume (Å^3^) | 91840±10000 | | |  | | 90820±10000 | 93520±10000 |  |  |  |
|  | Dry volume calculated from sequence (Å^3^) ‡ | ~54154 | | | ~54154 | | ~54154 | ~54154 |  |  |  |
| Molecular-mass determination | | | | | | |  |  |  |  |  |
|  | Molecular mass *M*_r_ (kDa) [from *Porod invariant*] | 41±5 | | |  | | 41±5 | 42±5 |  |  |  |
|  | Molecular mass *M*_r_ (kDa) [from *excluded volume*] | 46±5 | | |  | | 45±5 | 47±5 |  |  |  |
|  | Calculated monomeric *M*_r_ (kDa) [from *sequence*]* | ~44.7 | | | ~44.7 | | ~44.7 | ~44.7 |  |  |  |
|  | |  | | |  | |  |  |  |  |  |
| N-Cbl variant collection parameters | | ***pTyr371-Cbl*** | | | ***Y368F*** | | ***M222E*** |  |  |  |  |
|  | Concentration range (mg ml^-1^) | 0.5-10 | | | 0.5-10 | | 0.5-10 |  |  |  |  |
| Structural parameters | | | | |  | |  |  |  |  |  |
|  | *I*(0) (relative) [from *P*(*r*)] | 7699±15 | | | 6802±10 | | 7359±10 |  |  |  |  |
|  | *R*_g_ (Å) [from *P*(*r*)] | 28±1 | | | 24±1 | | 24±1 |  |  |  |  |
|  | *I*(0) (relative) [from Guinier] | 7726±15 | | | 6813±10 | | 7433±10 |  |  |  |  |
|  | *R*_g_ (Å) [from Guinier] | 28±1 | | | 24±1 | | 24±1 |  |  |  |  |
|  | *D*_max_ (Å) | 92±10 | | | 70±5 | | 70±10 |  |  |  |  |
|  | Porod volume estimate (Å^3^) | 71225±5000 | | | 630622±5000 | | 71326±5000 |  |  |  |  |
|  | Dammif excluded volume (Å^3^) | 95610±10000 | | | 91330±10000 | | 95610±10000 |  |  |  |  |
|  | Dry volume calculated from sequence (Å^3^) ‡ | ~54154 | | | ~54154 | | ~54154 |  |  |  |  |
| Molecular-mass determination | | | | | | |  |  |  | |  |
|  | Molecular mass *M*_r_ (kDa) [from *Porod invariant*] | 42±5 | | | 40±5 | | 43±5 |  |  |  |  |
|  | Molecular mass *M*_r_ (kDa) [from *excluded volume*] | 48±5 | | | 46±5 | | 48±5 |  |  |  |  |
|  | Calculated monomeric *M*_r_ (kDa) [from *sequence*]* | ~44.7 | | | ~44.7 | | ~44.7 |  |  |  |  |
| * <http://web.expasy.org/compute_pi/>  ‡ <http://www.basic.northwestern.edu/biotools/proteincalc.html> | | | | | | |  |  |  | |  |
|  | | | | | | |  |  |  | |  |
|  |  |  | | |  | |  |  |  |  |  |
|  |  |  | | |  | |  |  |  |  |  |
